# The association between emotional intelligence and burnout among Chinese nurses: a cross-sectional study

**DOI:** 10.3389/fpubh.2026.1803470

**Published:** 2026-04-13

**Authors:** Yunfeng Pan, Hongjuan Huang, Cuiyun Fang, Lei Sun, Wei Fan

**Affiliations:** Liyang People's Hospital, Changzhou, China

**Keywords:** burnout, Chinese, cross-sectional study, emotional intelligence, nurse

## Abstract

**Background:**

Nurse burnout is a widespread global occupational health challenge in healthcare. However, the association between emotional intelligence (EI) and burnout remains relatively under-investigated within China’s collectivist cultural context. What is the magnitude and direction of the association between EI and burnout among nurses in Chinese tertiary hospitals?

**Methods:**

A cross-sectional study was conducted among 308 eligible nurses from a Chinese tertiary hospital, using the Wong and Law Emotional Intelligence Scale (WLEIS) and Maslach Burnout Inventory-Human Services Survey (MBI-HSS). Data were analyzed using Pearson’s correlation analysis and multiple linear regression analysis.

**Results:**

Participants had moderate-to-high EI levels, with a mean total WLEIS score of 82.24 ± 15.06. The overall prevalence of clinically significant burnout was 65.3%, with 58.44% of participants reporting high emotional exhaustion, 48.05% high depersonalization, and 42.86% low personal accomplishment. EI was significantly inversely correlated with emotional exhaustion (*r* = −0.35, *p* < 0.01) and depersonalization (*r* = −0.23, *p* < 0.01), and significantly positively correlated with personal accomplishment (*r* = 0.50, *p* < 0.01). Furthermore, EI was independently associated with all three burnout dimensions (*β* = −0.39, −0.28, 0.53; *R*^2^ = 0.20, 0.16, 0.41; all *p* < 0.01).

**Conclusion:**

EI is significantly associated with all three burnout dimensions among Chinese tertiary hospital nurses, providing preliminary evidence for EI-focused burnout mitigation strategies and related research in the Chinese context.

## Introduction

Nurse burnout, a critical global challenge in healthcare systems (1), is characterized by three core dimensions: emotional exhaustion, depersonalization, and reduced personal accomplishment (2). Epidemiological data highlight the widespread prevalence of burnout among nurses. Cross-national studies indicate that approximately 30.0% of nurses globally experience burnout symptoms (3). Notably, burnout rates vary significantly across countries and regions due to professional differences, geographic factors, and measurement tools (4, 5). The prevalence of nursing burnout reaches 68% among Chinese nurses (6), compared with 62% among American nurses (7). Lower burnout prevalence rates are reported in other countries: 42% in England, 30% in the Philippines, 29% in Sweden, and 10% in the Netherlands (8, 9). Furthermore, the crisis has intensified during the COVID-19 pandemic (10, 11). Contributing factors include organizational stressors such as heavy workloads, long shifts, and inadequate managerial support, alongside individual challenges like insufficient recognition of contributions (12–14). Systemic issues within the Chinese healthcare system, including rigid hierarchical structures and inequities in employment contracts, further exacerbate stressors, disproportionately affecting junior and tertiary-level nurses (15, 16). The consequences of burnout extend beyond individual well-being, increasing risks of depression, insomnia, and cardiovascular disorders among nurses (17). Organizations face heightened turnover, medical errors, and compromised patient safety, including elevated rates of hospital-acquired infections (18). In response to this public health challenge, China’s National Health Commission has issued national policies requiring medical institutions to strengthen nurses’ mental health management and prevent occupational burnout. However, most existing burnout-mitigation measures in domestic hospitals focus on generalized strategies, including scheduling optimization, psychological counseling, and workload adjustment, with limited effectiveness in reducing burnout among frontline nurses. Two core gaps motivate this study: the persistently high burnout prevalence among Chinese tertiary hospital nurses and the limited efficacy of existing generalized mitigation strategies, highlighting an urgent need to identify targeted, evidence-based factors associated with lower burnout risk in this frontline population.

Emotional intelligence (EI), defined as the capacity to perceive, regulate, and apply emotions adaptively, has emerged as a critical competency for nurses (19, 20). The core components of EI: self-awareness, emotional regulation, empathy, and social skills enable nurses to navigate high-stress clinical environments, resolve interpersonal conflicts, and foster therapeutic patient relationships (21, 22). Existing correlational research has established an association between higher EI and greater job satisfaction (23). Similarly, existing studies have identified a significant negative association between higher EI and burnout vulnerability among nursing populations, with evidence showing that higher EI levels are linked to lower emotional exhaustion and depersonalization, as well as higher personal accomplishment (24–27). This correlation is hypothesized to link to nurses’ perceived control over workplace stressors, especially in settings requiring sustained emotional labor, such as oncology and long-term care units. EI-related practices in clinical nursing in China are mostly limited to fragmented, non-standardized communication skills training in daily work, with no systematic, culturally adapted EI capacity-building programs or unified implementation guidelines for burnout prevention developed for frontline nurses (28). This gap is particularly pronounced for tertiary hospital nurses, who face sustained heavy workloads and high emotional labor demands (29).

Cultural and organizational contexts influence the association between EI and burnout (30). In China’s collectivist healthcare systems, where interpersonal harmony is emphasized, EI may have a unique association with burnout mechanisms (31). However, existing literature predominantly focuses on Western populations, leaving a limited understanding of EI-burnout dynamics in Chinese nursing settings. This cross-sectional study focuses on nurses from a tertiary hospital in Liyang City, Changzhou, Jiangsu Province, in eastern China. This group has been underrepresented in previous regional research. Against this critical research gap, this study centers on the following core research question: What is the association between EI and burnout among nurses in Chinese tertiary hospitals? To rigorously address this core research question, the primary objective of this cross-sectional study is to quantify the aforementioned associations between EI and each burnout dimension, and to provide preliminary evidence for the development of culturally adapted occupational health strategies targeting nurse burnout in the Chinese clinical setting. The findings of this study will contribute to the evidence on the EI-burnout association within the collectivist cultural context of Chinese nursing.

## Method

### Study design

This cross-sectional study explored the association between EI and burnout among Chinese clinical nurses, conducted in strict accordance with the Strengthening Reporting of Observational Studies in Epidemiology (STROBE) for observational studies. The completed STROBE checklist is provided in the supplementary material. The study was performed at Liyang People’s Hospital, a public tertiary teaching hospital in Jiangsu Province, China, with 1,100 licensed beds, 1,612 staff, and 40 clinical/technical departments. This study adopted a positivist research philosophy, focusing on quantifiable observations of variable associations, consistent with cross-sectional study norms. Measures to mitigate bias included anonymous questionnaire design to reduce social desirability bias, validated standardized scales to minimize measurement bias, and pre-specified confounder adjustment to control for confounding bias.

### Participants

This study enrolled registered nurses working at the hospital. Inclusion criteria included: holding a valid nursing license in China; working in direct patient-care roles for ≥6 months; and providing informed consent. Exclusion criteria were: long-term leave (≥3 months due to illness or other reasons); engaged in full-time administrative or management work with no direct patient-care responsibilities; and refusal to participate. All eligible nurses in the hospital were invited to participate via convenience sampling, with no stratified sampling applied.

### Data collection

Recruitment and data collection were conducted between January 6 and January 20, 2025. All eligible nurses meeting the inclusion criteria were invited to participate via the hospital’s internal nursing work platform. We posted the official study introduction, a clear statement of voluntary participation, and the anonymous questionnaire link on the platform, with the invitation pushed to nurses who met the pre-specified inclusion criteria. An anonymous online questionnaire was administered via Questionnaire Star, a widely used, validated digital survey platform for academic research in China. Participants accessed the survey through the unique link in the platform notice, and no personal identifiable information (including name, job number, and department) was collected in the questionnaire to guarantee complete anonymity of all participants. The questionnaire consisted of three sections: demographic characteristics assessment, EI measurement, and nurse burnout measurement. Two automated reminders were sent to non-respondents via the internal nursing work platform on the 7th and 14th day of the data collection period, respectively. The study design and data collection procedures were consistent with validated methods for nursing burnout research (32).

### Measurement instruments

EI: EI was assessed using the Wong and Law Emotional Intelligence Scale (WLEIS) (33). This 16-item self-report scale measures four core domains of EI: self-emotion appraisal, others’ emotion appraisal, use of emotion, and regulation of emotion. WLEIS was selected for this study for three key reasons. First, it has been widely validated and used in Chinese nursing populations. It demonstrates good reliability and cultural adaptability (34). Second, it is a concise, self-administered tool. With a low response burden, it is suitable for large-sample online surveys (35). Third, compared with performance-based tools, WLEIS has better predictive validity for workplace outcomes, including burnout in nursing populations (36). Items were scored on a 7-point Likert scale (1 = strongly disagree to 7 = strongly agree), with total scores ranging from 16 to 112. Higher scores indicated higher EI. The scale showed excellent internal consistency in this study, with a Cronbach’s *α* of 0.941.

Nurse burnout: Nurse burnout was measured using the Maslach Burnout Inventory–Human Services Survey (MBI-HSS) (37). Official copyright approval for the use of the MBI-HSS was obtained from the copyright holder (Mind Garden, Inc.). The authorization document is provided in the supplementary material. This 22-item scale assesses three dimensions of burnout: emotional exhaustion (9 items), depersonalization (5 items), and personal accomplishment (8 items). Items use a 7-point frequency scale (0 = never, 6 = every day). Higher scores for emotional exhaustion and depersonalization, and lower scores for personal accomplishment, indicate more severe burnout. Burnout levels were categorized as follows: high, defined by a score of emotional exhaustion ≥ 27, depersonalization ≥10, personal accomplishment ≤33; moderate, indicated by emotional exhaustion 19–26, depersonalization 6–9, personal accomplishment 34–39; and low, characterized by emotional exhaustion ≤18, depersonalization ≤5, personal accomplishment ≥40. Significant burnout was defined as an emotional exhaustion score ≥ 27 and/or a depersonalization score ≥ 10. The MBI-HSS showed acceptable internal consistency in this study, with a Cronbach’s *α* coefficient of 0.824. The subscales also showed satisfactory reliability: Cronbach’s α was 0.928 for emotional exhaustion, 0.820 for depersonalization, and 0.897 for personal accomplishment.

### Ethical considerations

The study received full ethical approval from the Research Ethics Committee of Liyang People’s Hospital (Ethics Approval No. 2025041). All procedures were performed in accordance with the Declaration of Helsinki. Participation was entirely voluntary. Electronic informed consent was obtained from all participants prior to survey completion. Anonymity of all participants was guaranteed throughout the study. Confidentiality of all collected data was strictly maintained. All participants were informed of their right to withdraw from the study at any time without penalty.

### Statistical analysis

Based on a correlation coefficient of 0.5 between EI and nurse burnout reported in prior relevant studies (25), a minimum sample size of 37 was calculated using PASS 15.0 software (two-sided *α* = 0.05, statistical power = 0.90). Accounting for a 50% non-response rate common in online nurse surveys, the pre-specified target sample size was 74. The final 308 participants included in the analysis far exceeded this threshold, ensuring robust statistical power for all core analyses of this study. Descriptive statistics were used to summarize participants’ demographic variables, EI, and burnout scores. Continuous variables were presented as mean ± standard deviation (SD). Categorical variables are presented as frequencies and percentages. No missing data were observed in the final analysis, as all core questionnaire items were set as mandatory responses. Pearson’s correlation coefficients were calculated to assess the bivariate associations between EI and burnout dimensions. Multiple linear regression models were constructed to evaluate the independent association between EI and burnout, adjusting for confounders. All analyses were conducted using IBM SPSS Statistics version 26.0. A two-sided *p*-value < 0.05 was considered statistically significant.

## Results

### Demographic characteristics

Of 623 eligible nurses, 308 completed the survey, with a response rate of 49.4%. Participants were predominantly 26–35 years (37.99%), female (99.03%), with bachelor’s degrees (82.79%), married (80.52%), and 53.90% had one child. Most (38.64%) had ≥21 years of clinical work experience, 41.56% held intermediate technical ranks, and 66.88% worked night shifts. Detailed distributions of all demographic variables are presented in Table 1.

**Table 1:** Demographic characteristics of enrolled nurse participants (*N* = 308).

Variable	*N* (%)
Age (years)	
≤25	22 (7.14%)
26–35	117 (37.99%)
36–45	90 (29.22%)
46–55	75 (24.35%)
≥56	4 (1.30%)
Gender	
Male	3 (0.97%)
Female	305 (99.03%)
Education	
Junior college	51 (16.56%)
Bachelor	255 (82.79%)
Master	2 (0.65%)
Marital status	
Unmarried	50 (16.23%)
Married	248 (80.52%)
Divorce	9 (2.92%)
Others	1 (0.32%)
Existence of children	
0	63 (20.45%)
1	166 (53.90%)
2	75 (24.35%)
≥3	4 (1.30%)
Working age	
≤5 years	37 (12.01%)
6–10 years	66 (21.43%)
11–15 years	57 (18.51%)
16–20 years	29 (9.42%)
≥21 years	119 (38.64%)
Technical rank	
Internship	4 (1.30%)
Junior	82 (26.62%)
Intermediate	128 (41.56%)
Senior	94 (30.52%)
Night shift	
Yes	206 (66.88%)
No	102 (33.12%)

### Descriptive statistics for EI and burnout

The mean WLEIS score was 82.24 ± 15.06, indicating moderate-to-high EI levels among participants. The overall MBI-HSS score was 78.86 ± 18.45. Subscale analysis showed a high burnout prevalence: 58.44% had high emotional exhaustion, 48.05% had high depersonalization, and 42.86% had low personal accomplishment. The overall prevalence of burnout among participants in this study was 65.3%. Detailed scores of the two scales are summarized in Table 2.

**Table 2:** Total and subscale scores of the WLEIS and MBI-HSS.

Variable	Mean + SD/*N* (%)
WLEIS	82.24 ± 15.06
MBI-HSS	78.86 ± 18.45
Emotional exhaustion	31.06 ± 12.85
Low burnout	53 (17.21%)
Moderate burnout	75 (24.35%)
High burnout	180 (58.44%)
Depersonalization	10.99 ± 6.05
low burnout	0 (0.00%)
Moderate burnout	160 (51.95%)
High burnout	148 (48.05%)
Personal accomplishment	36.81 ± 12.01
Low burnout	118 (38.31%)
Moderate burnout	58 (18.83%)
High burnout	132 (42.86%)

SD, standard deviation; WLEIS, Wong and Law Emotional Intelligence Scale; MBI-HSS, Maslach Burnout Inventory-Human Services Survey.

### Correlation between EI and burnout dimensions

As depicted in Table 3, EI demonstrated significant correlations with each dimension of burnout. Pearson correlation analysis revealed a moderate inverse relationship between EI and emotional exhaustion (*r* = −0.35, *p* < 0.01) and depersonalization (*r* = −0.23, *p* < 0.01), as well as a strong positive correlation with personal accomplishment (*r* = 0.50, *p* < 0.01).

**Table 3:** Correlation between emotional intelligence and each burnout dimension.

Variable	Emotional intelligence	Emotional exhaustion	Depersonalization	Personal accomplishment
Emotional intelligence	1	−0.35^**^	−0.23^**^	0.50^**^
Emotional exhaustion	−0.35^**^	1	0.71^**^	−0.27^**^
Depersonalization	−0.23^**^	0.71^**^	1	−0.23^**^
Personal accomplishment	0.50^**^	−0.27^**^	−0.23^**^	1

** *p* < 0.01.

### Multivariable linear regression analysis

Multiple linear regression models were constructed to evaluate the independent association between EI and each dimension of nurse burnout, after adjusting for demographic covariates. As shown in Table 4, EI was independently and negatively associated with emotional exhaustion (*β* = − 0.39, *p* < 0.01; *R*^2^ = 0.20) and depersonalization (*β* = −0.28, *p* < 0.01; *R*^2^ = 0.16). EI was independently and positively associated with personal accomplishment (*β* = 0.53, *p* < 0.01; *R*^2^ = 0.41). These findings indicate that higher EI is associated with lower emotional exhaustion, lower depersonalization, and higher personal accomplishment among enrolled nurses.

**Table 4:** Multivariable linear regression analysis of the association between emotional intelligence and each burnout dimension.

Variable	Unstandardized coefficients		Standardized coefficients		*p*-value	*R*^2^	*F*	95% confidence interval for B	
	*B*	SE	*β*	*t*				Lower bound	Upper bound
Emotional exhaustion	−0.33	0.05	−0.39	−7.19	*p* < 0.01	0.20	8.31	−0.42	−0.24
Depersonalization	−0.11	0.02	−0.28	5.04	*p* < 0.01	0.16	6.47	−0.16	−0.07
Personal accomplishment	0.42	0.04	0.53	11.41	*p* < 0.01	0.41	22.63	0.35	0.50

## Discussion

This cross-sectional study aimed to explore the association between EI and burnout among Chinese tertiary hospital nurses. This study identified significant associations between EI and all three dimensions of burnout measured by the MBI-HSS among Chinese nurses. Higher EI scores were significantly correlated with lower emotional exhaustion and depersonalization, and higher personal accomplishment, consistent with previous reports (25, 27).

The demographic profile of our cohort provides critical context for interpreting the association between EI and burnout observed in this study. Participants were predominantly female (99.03%), which aligns with national nursing surveys in China showing male nurses account for only 3% of the total nursing workforce (38), a gender imbalance driven by long-standing traditional occupational and social norms. 38.64% of participants had 21 or more years of clinical experience, and 30.52% held senior nursing titles. The high burnout prevalence observed among these seasoned nurses indicates that organizational factors, rather than individual resilience alone, are associated with burnout in this population, which aligns with prior research identifying systemic inequities in Chinese healthcare, including disparities in employment contracts and career advancement opportunities (39). Junior nurses with non-tenure-track contracts are associated with higher occupational stress due to limited career stability, which may alter the magnitude of the association between EI and burnout regardless of EI levels (40). Most participants were married (80.52%) and worked regular night shifts (66.88%). In the context of China’s family-centered cultural norms, many nurses carry dual responsibilities of clinical work and family care, including childcare and elder care. The combination of heavy clinical workload, night shift work, and family obligations may reduce available emotional resources and may weaken the association between higher EI and lower burnout risk.

To contextualize our core findings on the EI-burnout association, we systematically compared our core measurements of EI and burnout with published data from Asian, Western, and international nursing cohorts. The mean total WLEIS score in this study was 82.24 ± 15.06, indicating moderate-to-high EI among Chinese tertiary hospital nurses. This score was consistent with prior multicenter research in Chinese clinical nurses, which reported a mean WLEIS score of 84.21 ± 13.48 (41), and was also comparable to scores reported in Japanese hospital nurses (42) and South Korean general hospital nurses (43). The consistent moderate-to-high EI levels across East Asian nursing cohorts provide a uniform baseline for interpreting the EI-burnout association observed in our study. For Western nursing populations, direct quantitative comparisons of WLEIS scores are limited by heterogeneous EI measurement practices: the WLEIS is predominantly validated and used in Asian studies, while European and North American nursing cohorts primarily use alternative validated tools, including the Mayer–Salovey–Caruso Emotional Intelligence Test and Trait Emotional Intelligence Questionnaire (44, 45). Limited cross-cultural data showed Western nurses report higher scores in emotion regulation and utilization dimensions, with no significant cross-cultural difference in the others’ emotion appraisal dimension (46), which may explain differences in the magnitude of the EI-burnout association between Eastern and Western cohorts.

For burnout dimensions, the overall prevalence of clinically significant burnout in our cohort was 65.3%. This rate is consistent with the 62% overall burnout prevalence among American nurses reported in prior national research (7). For specific subscales, the mean emotional exhaustion score was 31.06 ± 12.85, with high emotional exhaustion identified in 58.44% of participants. This percentage was significantly higher than the 48.3% rate in the American national cohort (7), the 38.5% rate among European nurses (47), and the 36.4% rate reported in a meta-analysis of Asian oncology nurses (48). For depersonalization, the mean score was 10.99 ± 6.05, with high depersonalization found in 48.05% of participants. This prevalence is notably higher than the 32.1% high depersonalization rate in American nurses (7), 28.6% in European nurses (47), 41.3% in Japanese nurses (49), and 39.7% in Iranian nurses (50). Notably, no participants in our study had low depersonalization scores. This is likely due to Chinese cultural norms that strongly emphasize interpersonal harmony in clinical settings: nurses are expected to maintain empathy and respect in patient and colleague interactions, and open expression of emotional detachment is widely discouraged, while heavy clinical workloads simultaneously increase the risk of moderate-to-high depersonalization. For personal accomplishment, the mean score was 36.81 ± 12.01, with low personal accomplishment detected in 42.86% of participants. This percentage is notably higher than the 27.8% low personal accomplishment rate reported in American nurses (7) and 25.3% in European nurses (47). Results are more consistent with other Asian populations, with similar prevalence reported in Malaysian nurses and Iranian nurses (50, 51). The overall higher burnout prevalence in our cohort highlights the severity of occupational stress among Chinese tertiary hospital nurses, and provides a critical context for interpreting the observed association between EI and burnout.

A significant negative correlation between EI and the emotional exhaustion and depersonalization dimensions of burnout, as well as a significant positive correlation between EI and personal accomplishment, was observed in this study. For emotional exhaustion, the observed inverse association between EI and this dimension may be contextualized by existing evidence on the correlation between EI and adaptive emotional regulation capacities: prior studies have identified a significant association between higher EI and stronger adaptive emotion regulation skills, which are in turn correlated with lower emotional depletion related to long-term high-intensity clinical work and frequent emotionally charged patient interactions (24, 52). For depersonalization, the observed inverse association between EI and this burnout dimension aligns with the core domains measured by the WLEIS, including empathy, others’ emotion appraisal, and social skills. Existing literature has reported significant correlations between these EI-related competencies, better maintenance of therapeutic patient relationships, lower emotional detachment, and lower depersonalization risk in high-stress clinical settings (22, 53). This association is particularly relevant in China’s collectivist work environment, where interpersonal harmony is a core cultural value (54). In such settings, higher EI is associated with better capacity to manage complex interpersonal dynamics and resolve workplace conflicts, which aligns with the correlation between higher EI and lower burnout risk observed in this study (55). Cultural values in China also shape the association between EI and burnout dimensions. Collectivist norms are associated with a stronger link between EI and personal accomplishment (56, 57). Professional accomplishment among Chinese nurses is often closely tied to team and departmental performance rather than individual achievement alone. The EI domains measured in this study, including the use of emotion and others’ emotion appraisal, are correlated with teamwork, communication, and conflict resolution skills in nursing settings. These skills have been linked to nurses’ contributions to collective team goals, which is in turn correlated with higher personal accomplishment (58).

The inverse association between EI and emotional exhaustion, and the positive association between EI and personal accomplishment, align with theoretical models that describe the correlation between EI, adaptive emotion regulation, interpersonal skills, and stress resilience in occupational settings (52). This dynamic is particularly relevant in Chinese nursing settings, where hierarchical hospital structures, prolonged work hours, and insufficient managerial support contribute to chronic stress (59). The observed high prevalence of emotional exhaustion (58.44%) underscores the urgency of addressing systemic challenges such as workload optimization and recognition of nursing contributions (60). In collectivist cultures, where interpersonal harmony and social cohesion are prioritized, the association between EI and burnout outcomes may be more pronounced (61). Prior studies have noted that in collectivist cultural contexts, EI is correlated with nurses’ capacity to navigate interpersonal expectations, foster therapeutic relationships, and maintain emotional balance in high-pressure clinical settings, which may contextualize the associations observed in our study (55).

Our study expands existing research by examining Chinese nurses, a population historically underrepresented in EI-burnout investigations. The strong positive correlation between EI and personal accomplishment (*r* = 0.50) exceeds effect sizes reported in Western nursing cohorts (27, 53). This suggests that cultural norms emphasizing collective achievement and professional efficacy may shape the strength of the EI-personal accomplishment association in Chinese contexts. The strong positive association between EI and personal accomplishment observed in this study may be contextualized by prior evidence, which has identified significant correlations between higher EI, more effective therapeutic patient relationships, and greater nursing job satisfaction (24). In China’s healthcare landscape, where nurse–patient communication is increasingly prioritized amid systemic reforms, empathy and social competence are correlated with higher perceived professional efficacy, which has been linked with higher personal accomplishment (62).

Global estimates suggest that approximately 30.0% of nurses experience burnout worldwide (3). The burnout rate in our study, as in many Western tertiary care settings, is significantly higher. This elevation stems from a heavier workload and greater emotional labor in tertiary hospitals, compared with primary care settings (63). Despite similarly high burnout rates, the underlying mechanisms differ markedly between China and Western countries (56). In China, burnout is associated with the interaction of collectivist norms and health system stressors. Related structural factors exacerbate chronic occupational stress and may weaken the magnitude of the association between EI and lower burnout levels (64). In Western settings, burnout is primarily associated with mismatches between individual values and systemic working conditions, such as conflicts between expectations for autonomy and work-life balance and realities of staffing shortages and high workload (65, 66). These cross-cultural differences highlight the need to interpret the EI-burnout association within specific cultural and systemic contexts.

In addition to emotional intelligence, multiple factors have been associated with nurse burnout worldwide. These factors can be categorized into workplace, individual, family, and socio-cultural domains. Major workplace factors include high workload, long working hours, interpersonal conflicts, unfair treatment, insufficient recognition, unfavorable work environment, and unresolved organizational issues (67, 68). Such conditions contribute to chronic occupational stress and may reduce the magnitude of the association between EI and burnout (25, 69). Individual and family factors, such as family care burden, financial pressure, unhealthy lifestyle, and mental health problems, are also associated with reduced emotional regulation ability and higher burnout vulnerability (70). Socio-cultural factors, including inadequate social support, collectivist cultural norms, and stigma toward mental health help-seeking, may further exacerbate the effects of occupational stress (56). The high burnout prevalence observed in this study likely reflects the combined influence of these multilevel factors rather than EI alone. These findings highlight that effective burnout prevention strategies need to address both individual EI capacities and broader systemic drivers of occupational stress.

While this study provides valuable insights into the association between EI and nurse burnout, several limitations must be acknowledged. First, this study used a non-experimental cross-sectional observational design, which cannot confirm the temporal sequence between EI and burnout or establish definitive causal inferences. Longitudinal cohort studies are needed to clarify their temporal dynamics, while randomized controlled trials of targeted EI interventions are required to verify causal relationships. Second, this study relied solely on self-reported scales for data collection, which may introduce social desirability bias. Future research should incorporate objective physiological markers of burnout, combined with multi-method approaches including clinical observation, case studies, and mixed-methods designs, to corroborate self-reported findings. Third, the study sample was recruited from a single tertiary hospital in China, restricting generalizability. Replication studies across diverse settings, including urban and rural hospitals, are needed to enhance the robustness of the conclusions. Fourth, we did not collect department-specific data from enrolled participants during data collection. We are therefore unable to perform subgroup analyses exploring the impacts of departmental culture, workload, and practice environment on the EI-burnout association. Future studies are recommended to collect detailed department-level data to address this gap. Fifth, the study included almost exclusively female participants, which may limit the generalizability of findings to male nurses. Lastly, the study did not examine potential moderating variables like organizational culture or leadership styles, which may shape the EI-burnout relationship in complex ways. Future cross-cultural and culturally focused studies are encouraged to explore how cultural values, gender distribution, family responsibilities, and systemic factors interact with EI to influence nurse burnout. Addressing these gaps through rigorous study designs will advance the understanding of contextual factors influencing burnout resilience in nursing populations.

## Conclusion

This study identified significant associations between EI and all three burnout dimensions measured by MBI-HSS among Chinese tertiary hospital nurses, with higher EI scores correlated with lower emotional exhaustion and depersonalization, and higher personal accomplishment. Contextualized within China’s collectivist culture and healthcare system, these findings indicate EI is a relevant individual factor linked to lower burnout levels and a potential target for hospital occupational health initiatives. Based on these results, we propose two preliminary practice recommendations: developing culturally adapted, standardized EI training programs for nurses in Chinese tertiary hospitals, and integrating targeted EI capacity building into in-service nursing education and comprehensive burnout prevention strategies addressing systemic workplace stressors. The effectiveness of EI-targeted interventions on burnout outcomes requires further empirical validation, and multi-center longitudinal studies and randomized controlled trials with objective burnout indicators are needed to inform evidence-based, context-specific burnout mitigation strategies for Chinese nurses.

## Data Availability

The original contributions presented in the study are included in the article/supplementary material, further inquiries can be directed to the corresponding author.
